# Ocean acidification modulates material flux linked with coral calcification and photosynthesis

**DOI:** 10.1038/s41598-025-30818-4

**Published:** 2025-12-12

**Authors:** David A. Armstrong, Conall McNicholl, Keisha D. Bahr

**Affiliations:** 1 Harte Research Institute, Texas A&M University-Corpus Christi, Corpus Christi, TX USA; 2 Hawaiʻi Institute of Marine Biology, University of Hawaiʻi at Mānoa, Kāneʻohe, HI USA

**Keywords:** pCO_2_, Aragonite saturation state (Ω), *Montipora capitata*, *Pocillopora acuta*, Carbonate chemistry, Concentration boundary layer, Microsensors, Coral reefs, Animal behaviour, Animal physiology, Climate-change impacts, Marine biology, Marine chemistry, Fluid dynamics

## Abstract

Coral reefs are essential for the foundation of marine ecosystems. However, ocean acidification (OA), driven by rising atmospheric carbon dioxide (CO_2_) threatens coral growth and biological homeostasis. This study examines two Hawaiian coral species—*Montipora capitata* and *Pocillopora acuta* to elevated pCO_2_ simulating OA. Utilizing pH and O_2_ microsensors under controlled light and dark conditions, this work characterized interspecific concentration boundary layer (CBL) traits and quantified material fluxes under ambient and elevated pCO_2_. The results of this study revealed that under increased pCO_2_, *P. acuta* showed a significant reduction in dark proton efflux, followed by an increase in light O_2_ flux, suggesting reduced calcification and enhanced photosynthesis. In contrast, *M. capitata* did not show any robust evidence of changes in either flux parameters under similar increased pCO_2_ conditions. Statistical analyses using linear models revealed several significant interactions among species, treatment, and light conditions, identifying physical, chemical, and biological drivers of species responses to increased pCO_2_. This study also presents several conceptual models that correlate the CBL dynamics measured here with calcification and metabolic processes, thereby justifying our findings. We indicate that elevated pCO_2_ exacerbates microchemical gradients in the CBL and may threaten calcification in vulnerable species such as *P. acuta*, while highlighting the resistance of *M. capitata*. Therefore, this study advances our understanding of how interspecific microenvironmental processes could influence coral responses to changing ocean chemistry.

**Supplementary Information:**

The online version contains supplementary material available at 10.1038/s41598-025-30818-4.

## Introduction

**Fig. 1:**
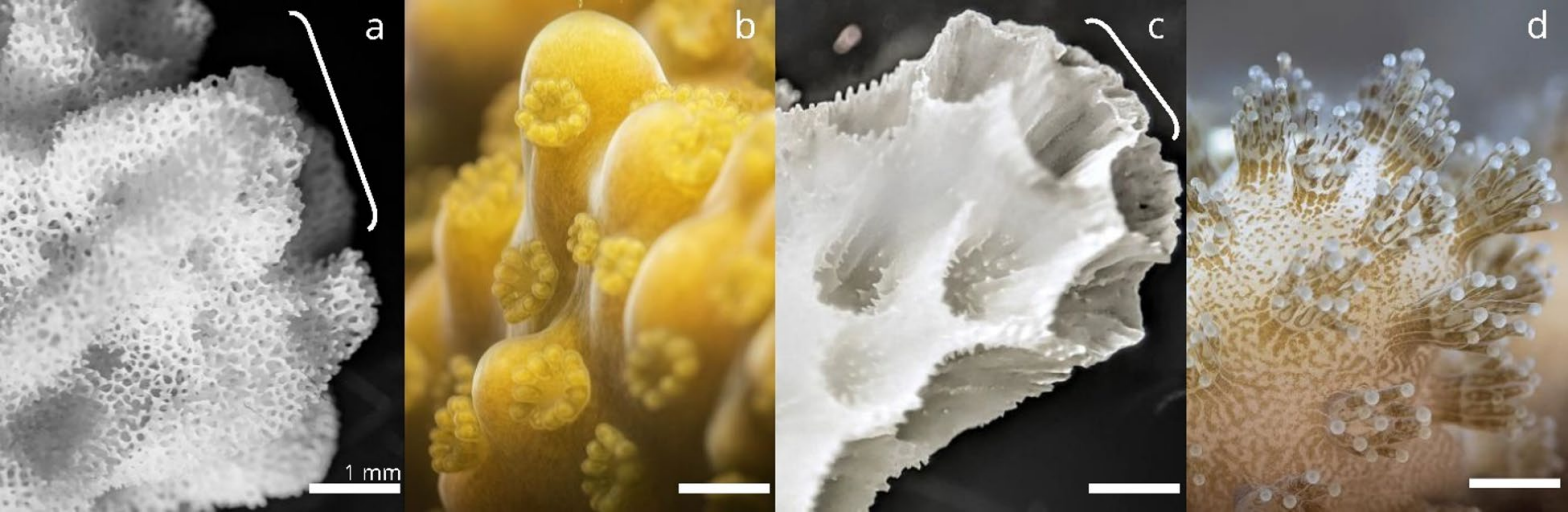
Stereoscope images of living corals in seawater or skeletal fragments. Shown from left to right, (**a**) *Montipora capitata* skeletal fragment (complex), with the white bracket indicating the zone of primary calcification (ZPC), **(b**) *M. capitata* tissue surface, **(c**) *Pocillopora acuta* skeletal fragment (robust) with ZPC indicated by a white bracket, and **(d**) *P. acuta* tissue surface. The white scale bar in each image is representative of ~ 1 mm.

 With rising atmospheric carbon dioxide (CO_2_) from anthropogenic activities, observations indicate decreasing ocean pH and aragonite saturation state (Ω_arag_) along with increasing the partial pressure of CO_2_ (pCO_2_), a process known as ocean acidification (OA)^1–3^. Future predictions (AR6; B.1.3; Scenario SSP5-8.5) for the years 2081–2100 show that surface pH levels of the North Pacific Ocean are ‘*virtually certain*’ to decrease by 0.28–0.29 units, resulting to a median value of 7.70 pH units^4^. This shift would increase hydrogen ion concentrations ([H^+^]) by nearly ~ 100%, following the standards presented by the IPCC (2022) in the year 2100^5^. Seawater chemistry is tightly linked to calcifying organisms ability to precipitate calcium carbonate (CaCO_3_) skeletons^1–3,5,6^. Such calcification has been observed in scleractinian corals under acidification at both the ecosystem^7–11^ and individual scales^12–16^. However, more importantly, responses are not uniformly shared among species^15–17^, and interact with environmental factors such as irradiance^13^ and seawater movement surrounding coral colonies^18,19^. Coral calcification under OA varies empirically, ranging from + 45% to −100% relative to ambient conditions, therefore, identifying the driver(s) of response is of the utmost importance for addressing the ongoing climate crisis^20–22^. To bridge this gap, interspecific responses show promise when considering morphology and the influence thereof on localized boundary layers (BL) at various depths from the surface of the coral^23,24^. This study aims to test this postulate through discrete measurements of proton and oxygen (O_2_) flux within the smallest BL of *Montipora capitata* and *Pocillopora acuta*, both classified as branching corals, but differ greatly in microtopography (Fig. 1).

Bulk advection barriers, where diffusion becomes the dominant mass-transfer process surrounding the coral, may act as an interspecific property that drives physical and chemical limitations to calcification. This study refers to this physical diffusive barrier as the concentration boundary layer (CBL), a thin, quiescent seawater layer surrounding the coral^18,25,26^. The CBL has been established in *M. capitata* (previously: *M. verrucosa*), localized to ~ 2.0 mm above the coral surface^27^ and shown in recent literature to be as small as ~ 0.1–0.2 mm thick in branching corals *Acropora cytherea*, *Pocillopora verrucosa*, and *Porites cylindrica*^25^. Several studies have explored CBL diffusion limitations using flow speed, calcifier taxa, morphology, and light/dark scenarios as proxies to explain the biological drivers of environmental change from OA^18,19,25,28,29^.

Across coral species, morphological comparisons such as branching or mounding^19^ and small-polyp or large-polyp corals driving CBL differences have been well studied^30,31^. Shown less frequently are the CBL dynamics between small-polyped coral species^25,31^. However, recent reports have highlighted ciliary vortices that disrupt the CBL in small-polyp coral species under slow flow^32,33^. Microtopographic differences influencing CBL dynamics, including corallite size and density among small-polyped species have only recently been established in Martins et al.^31^. For reference, *M. capitata* has corallites spaced ~ 25 polyps cm^− 2^ at ~ 2 mm in diameter, more sparse than those of *P. acuta*, which are ~ 70 polyps cm^− 2^ at ~ 1 mm in diameter (Fig. 1b, d)^34,35^. Morphologies within complex or robust groups have also not been compared frequently in CBL analysis, where perforate skeletons in *M. capitata* and imperforate skeletons in *P. acuta* may drive differences in the CBL (Fig. 1a, c). *Montipora capitata* has shown resistance to elevated pCO_2_ and several other environmental stressors, potentially due to plastic internal physiological mechanisms^13,17,34,36,37^. Conversely, *Pocillopora* spp. has shown variable responses, with some reports showing a 26% decrease in calcification under OA^12^. This study uses both species to characterize CBL characteristics linked to known OA sensitivity and the micromorphological constraints in localized environments where calcification is dominant.

Branching corals calcify rapidly at the distal ends of their branches, and their symbionts are least present in this region, termed the zone of primary calcification (ZPC; Fig. 1a, c)^23^. Within the ZPC, calcification may be less reliant on photosynthesis and more on diffusion^23^. The dissipation of calcification inhibitors (e.g., H^+^), the influx of substrates (e.g., Ca^2+^ and DIC), or the diffusion of gases such as O_2_^38^ could impose microchemical limitations on calcification^23,26^. Changes in ocean chemistry induced by OA may have severe negative consequences for diffusion-limited microenvironments at the ZPC^18,23,25,26,31^. Several biogeochemical hypotheses related to OA’s effects on coral calcification have been circulated, where the more prevalent adversity has been regarded as the depletion of [CO_3_^2−^] ions, a consequence of decreased seawater Ω_arag_^2,39,40^. Although not always considered, oceanic [H^+^] ions will simultaneously increase, and reduced proton efflux from the calcifying fluid (cf.) and overlying tissues may further limit coral calcification^26,41^. Supporting the latter, molecular reports have found no CO_3_^2−^ ion transporters in corals^42,43^, calcification shows a higher sensitivity to bicarbonate ion concentrations ([HCO_3_^−^]) over other DIC species^44^, and the ratio of [HCO_3_^−^]: [H^+^] explains coral calcification better compared to Ω_arag_^45^. The fundamental nature of OA’s interaction with calcification in corals remains poorly elucidated. This study advances our understanding of how proton flux is affected by increasing OA, an emerging ideology grounded in the biological interpretation of chemical limitations to coral calcification.

Measurements of flux within the CBL have previously been used as a proxy for characterizing the internal physiological responses to acidification. Comeau et al.^18^ showed that CBL pH measurements can provide indirect correlations with the internal chemistry of the cf., such as the pH_cf._ and Ω_cf._^18^. Hohn and Merico^46^ also reported increased leakage or diffusion through paracellular pathways connecting to the CBL, influencing the pH_cf._ and [CO_3_^2−^]. Venn et al.^47^ concluded that the immediate microenvironment influences pH_cf._ through its effects on the basal side of the calicodermis. Jokiel et al.^23^ proposed a two-cell model for the ZPC in which diffusion is primarily linked to exporting the calcification inhibitor H^+^ (protons), supporting Jokiel^26^ on the boundary layer limitations from proton flux under OA^23,26^. Therefore, CBL measurements at the ZPC of branching corals may provide insight into the internal physiological processes directly associated with rapid calcification, and how calcification may be impacted by the diffusion limitations imposed by OA.

Material flux, as defined by Fick’s first law of diffusion, states that the diffusion of materials occurs from high to low concentration areas. Proton flux at the ZPC is essential for coral calcification and for maintaining internal pH chemistry that supports the precipitation of aragonite^23,26^. However, under OA, proton flux from the boundary layer encounters a shallower diffusive gradient due to the increase in external [H^+^]^26^. Interspecific microtopographic attributes discussed previously may define flux conditions for material exchange within the boundary layer. O_2_ flux has been addressed in CBL analysis among most current reports^25,32,33^, but proton flux has not yet been examined through direct measurements in the ZPC of branching corals. Furthermore, interactions between O_2_ and proton flux in the immediate microenvironment at the ZPC have not been explored. This study fills this gap by characterizing the microchemical influence of acidified seawater chemistry on the CBL, identifying interspecific material flux limitations, and creating internal physiological assumptions based on external measures.

In this study, we examined the microenvironment of two Hawaiian coral species, *M. capitata* and *P. acuta*, under OA in both light and dark conditions. Using genotypically identical corals, this study compares control and elevated pCO_2_ (OA) treatments to assess interspecific responses. By simultaneously measuring pH and O_2_, we hypothesize that microchemical gradients at the ZPC would reveal interspecific physiological constraints on internal calcification under OA^19^. Building on previous work by Comeau et al.^18^ and Martins et al.^25,31^, this study further elucidates the microenvironmental factors that regulate coral calcification.

## Results

### Proton flux and CBL thickness

**Fig. 2:**
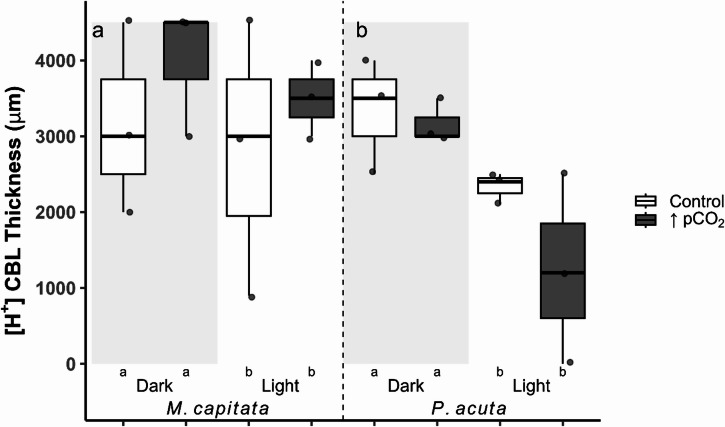
Boxplots of the proton ([H^+^]) boundary layer thickness (µm; Eqs. 1, 2) in both coral species, *Montipora capitata* (**a**) and *Pocillopora acuta* (**b**). Box color distinguishes treatment (Control = ambient, ↑pCO_2_ = ocean acidification), and the shaded region distinguishes light from dark conditions. Values are represented as individual profiles, with points, and the first and third quartiles are shown as boxes, with the median and whisker lines indicating the minimum and maximum. Significant differences are denoted as letters below the x-axis.

A linear mixed model (LMM) with genotype as the random factor revealed no significant interaction with genotypes (duplicated across treatments). This model was simplified, dropping genotype (as an individual ID and as a blocking factor for the tank effect) as a random factor. The proton CBL thickness (Eqs. 1, 2) differed significantly between light and dark conditions (three-way ANOVA, f = 5.04, df = 1, *p* = 0.036; Fig. 2a, b). However, there was no strong evidence to suggest that increased pCO_2_ treatment (three-way ANOVA, f = 0.02, df = 1, *p* = 0.876) and species (three-way ANOVA, f = 4.03, df = 1, *p* = 0.058) differed in proton CBL thicknesses (Fig. 2a, b). Residuals deviated from normality, however, a robust linear model subsequently produced the same significant levels.

**Fig. 3:**
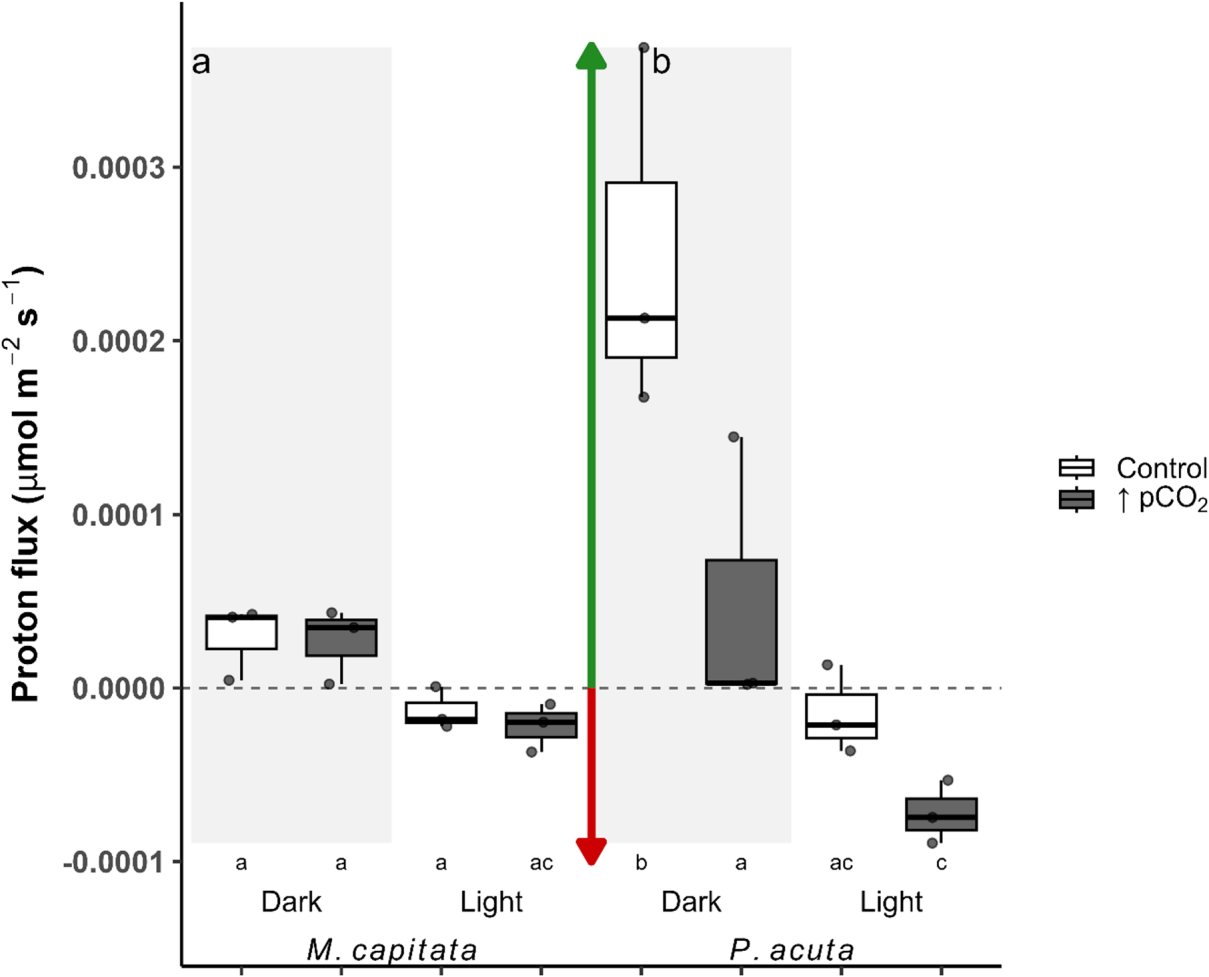
Boxplots of proton flux (µmol m^− 2^ s^− 1^; Eq. 3) in both coral species, *Montipora capitata* (**a**) and *Pocillopora acuta* (**b**). Box color distinguishes treatment (Control = ambient, ↑pCO_2_ = ocean acidification), and the shaded region distinguishes light from dark conditions. Values are individual flux values per profile, represented as points, and boxes are the first and third quartiles with the median and whisker lines indicating the minimum and maximum. Arrows indicate flux direction, the green arrow (positive values) is efflux from the coral surface, and the red arrow (negative values) is influx to the coral surface. Significance between groups was denoted by letters below the x-axis.

An LMM with genotype and tank interactions produced a singular fit for proton flux, random factors were dropped similarly to CBL thickness. The linear model with interactive effects (lowest AIC compared to the additive model) produced residuals that slightly deviated from normality, a Box-Cox Yeo-Johnson transformation (accounting for negative values) was applied to refit the model, and significance levels did not deviate (Fig. 3a, b). Pairwise comparisons with Bonferroni-adjusted p-values revealed significant differences among several levels of treatment, condition, and species (Fig. 3a, b; Table S1). Strong evidence for an isolated treatment effect to elevated pCO_2_ was observed only in *P. acuta* under dark conditions, where efflux decreased relative to group means (Fig. 3a; three-way interaction ANOVA, est. = 8.17e^− 5^, df = 16, *p* = 0.047). Compared across species and conditions, dark-driven proton efflux in control *P. acuta* differed significantly from all groups (Table S1). Elevated pCO_2_ showed strong evidence to support increased light-driven proton influx in *P. acuta* that differed from the control group *M. capitata* (Fig. 3a, b; three-way interaction ANOVA, est. = 8.13e^− 5^, df = 16, *p* = 0.049). In contrast, there was no strong evidence to suggest that the control groups differed in the light condition between species.

### O_2_ flux and CBL thickness

**Fig. 4:**
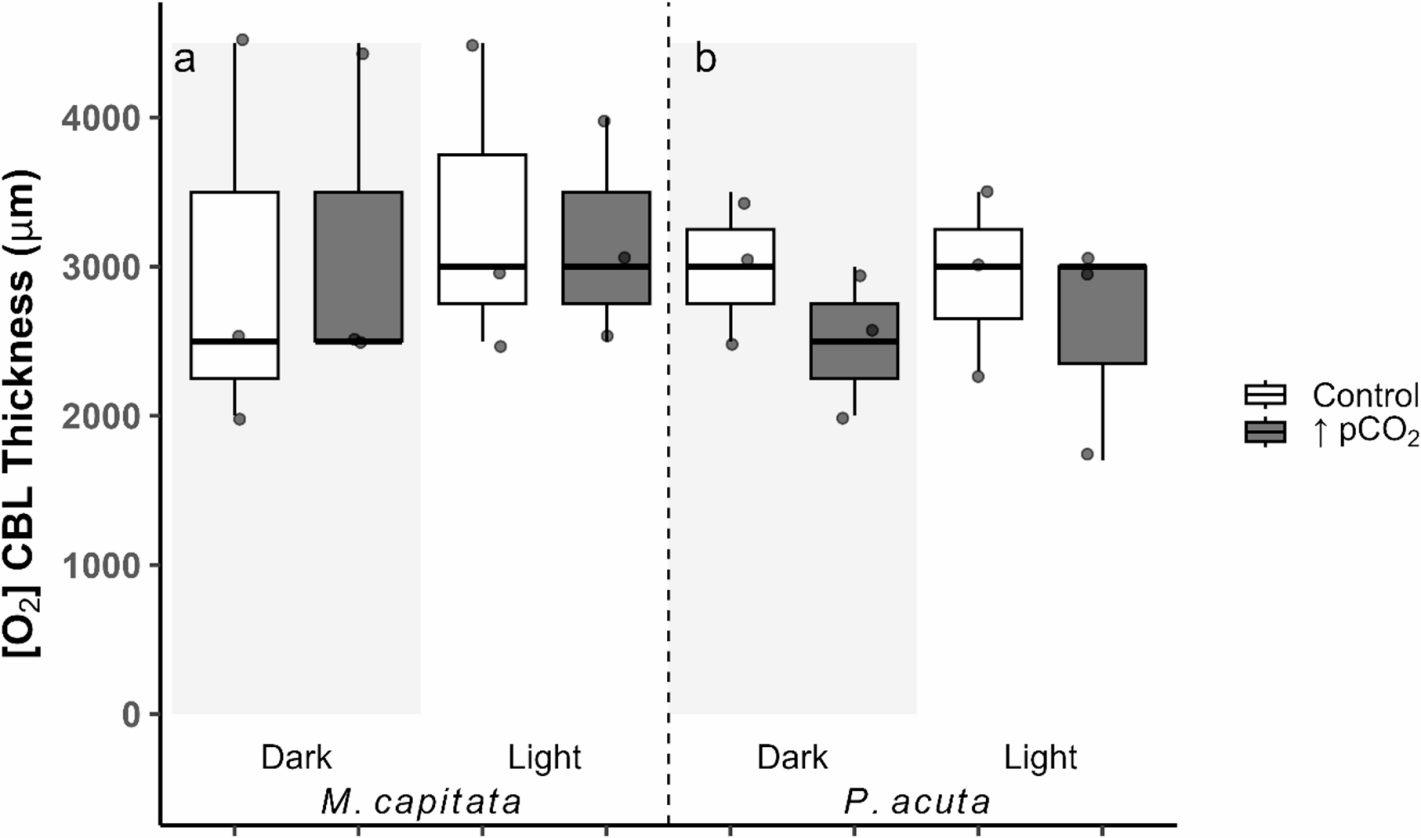
Boxplots of the oxygen ([O_2_]) boundary layer thickness (µm; Eqs. 1, 2) in both coral species, *Montipora capitata* (**a**) and *Pocillopora acuta* (**b**). Box color distinguishes treatment (Control = ambient, ↑pCO_2_ = ocean acidification), and the shaded region distinguishes light from dark conditions. Values are represented as individual profiles, with points, and the first and third quartiles are shown as boxes, with the median and whisker lines indicating the minimum and maximum.

The [O_2_] CBL thickness (Eqs. 1, 2) was assessed using a LMM, with genotype and tank as random factors. We did not find strong evidence to support differences in [O_2_] CBL thickness across species, treatment, and condition (Fig. 4a, b).

**Fig. 5:**
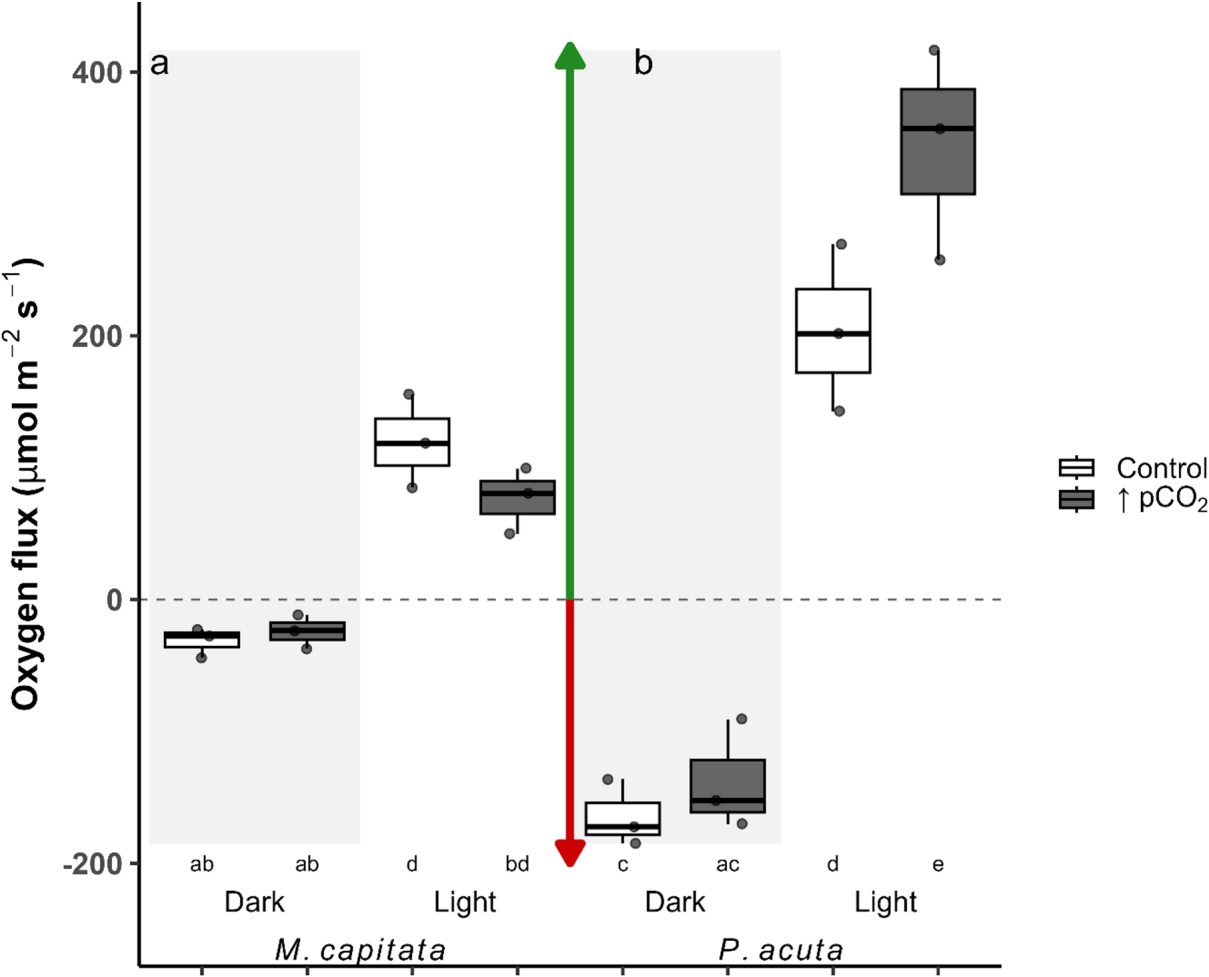
Boxplots of the oxygen flux (µmol m^− 2^ s^− 1^; Eq. 4) in both coral species, *Montipora capitata* (**a**) and *Pocillopora acuta* (**b**), box color distinguishes treatment (Control = ambient, ↑pCO_2_ = ocean acidification), and the shaded region distinguishes light from dark conditions. Arrows indicate flux direction, the green arrow (positive values) is efflux from the coral surface, and the red arrow (negative values) is influx to the coral surface. Values are represented as individual profiles, with points, and the first and third quartiles are shown as boxes, with the median and whisker lines indicating the minimum and maximum. Significance between groups was denoted by letters below the x-axis.

An LMM analysis of oxygen flux showed that genotype did not significantly contribute to explaining the data, therefore, similar in nature to the analysis of proton flux, this factor was omitted. The model with interactions had the lowest AIC score, and all p-values were calculated via pairwise comparisons with Bonferroni-adjusted values. On the treatment level comparison, there was strong evidence to support the effect of increased oxygen flux by ~ 40% change in *P. acuta* under light conditions (Fig. 5b; three-way interaction ANOVA, est. = −139, df = 16, *p* = 0.033). Between-species oxygen efflux under increased pCO_2_ in light conditions for the species *P. acuta* was significantly increased from all measures (Fig. 5a, b; Table S2). Dark oxygen influx significantly differed in the control group *P. acuta* compared to both treatment groups of *M. capitata*, whereas, under increased pCO_2,_ oxygen influx in *P. acuta* did not differ between the treatment groups of *M. capitata* (Fig. 5a, b; Table S2).

### Surface oscillations of pH_T_ and [O_2_]

**Fig. 6:**
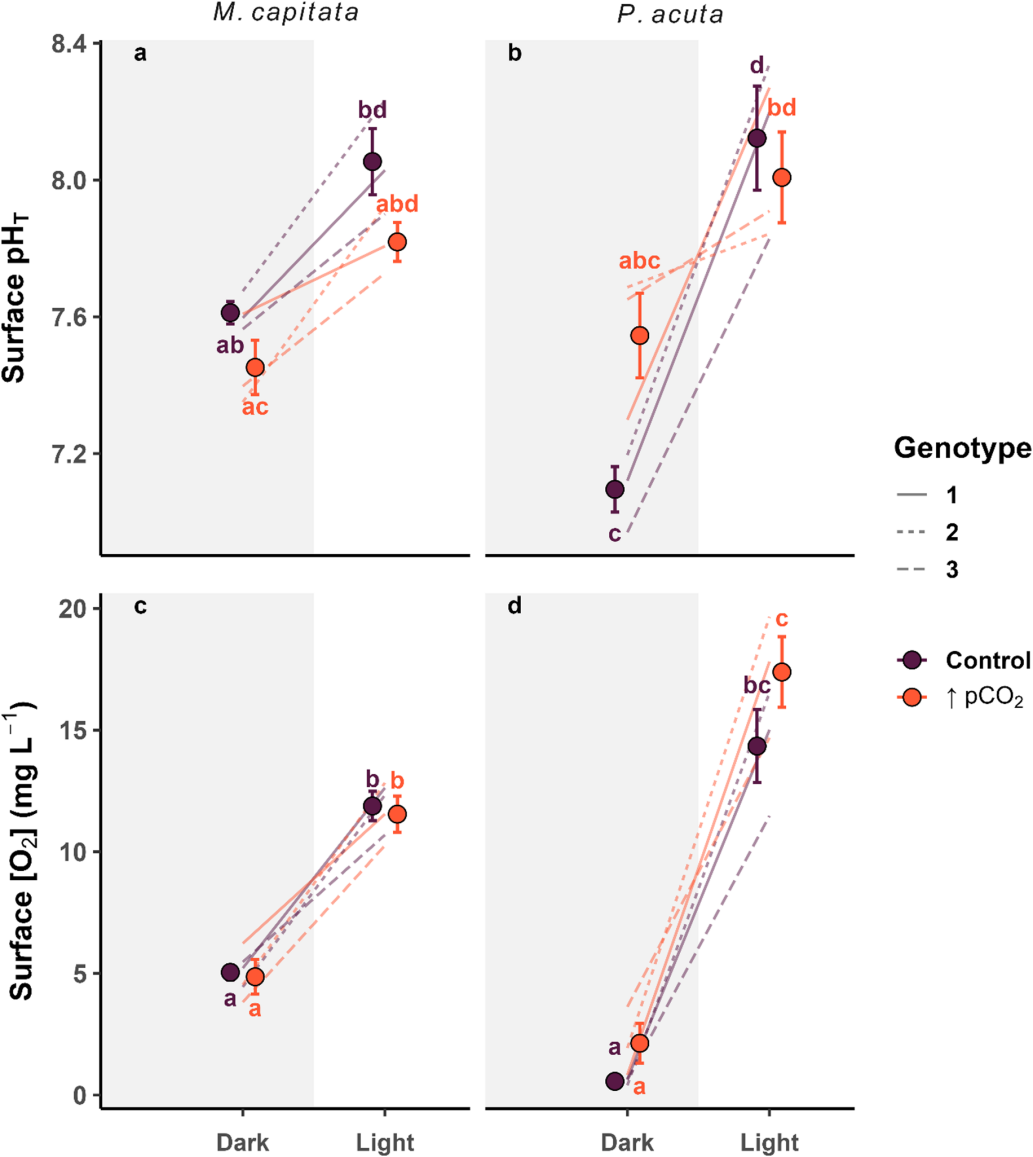
Slope dot plots comparing the mean ± SE (points) surface (0 μm) pH total (pH_T_; **a**, **b**) and concentrations of oxygen ([O_2_] mg L^− 1^; **c**, **d**) between dark and light conditions (shaded regions) for species *Montipora capitata* and *Pocillopora acuta* in response to treatment (Control = ambient, ↑pCO_2_ = ocean acidification). Letters adjacent to points indicate significantly different means with respect to the measure of either pH_T_ or [O_2_] mg L^− 1^. Any shared letters among groups indicate means that were not significantly different, where letters that do differ were resulting significant groups. Slope lines show individual genotypic variation relative to the means and are distinguished by line type.

An interaction-based LMM with genotype as a random factor and Bonferroni pairwise adjusted p-values on the surface pH_T_ values revealed strong evidence for light-driven pH_T_ in *P. acuta* in the control group but not under increased pCO_2_. The mean ΔpH_T_ = 1.02 for the control was nearly halved with a ΔpH_T_ = 0.54 for the increased pCO_2_ group (Fig. 6b; Table S3). Furthermore, the control groups surface pH_T_ of *P. acuta* in dark conditions was significantly lower than that of *M. capitata*, at pH_T_ = 7.10 ± 0.12 and pH_T_ = 7.61 ± 0.06 SD, respectively (Fig. 6a, b). There was no strong evidence to suggest that increased pCO_2_ significantly acidified the surface of the corals in both light and dark conditions (Fig. 6a, b; Table S3).

A second LMM with an identical design as the pH_T_ analysis on the [O_2_] levels provided strong evidence for light-driven changes in surface [O_2_] for both species, whereas treatment had no significant impact under increased pCO_2_ (Fig. 6c, d; Table S4). However, increased pCO_2_ significantly increased the mean [O_2_] by 5.9 mg L^− 1^ for *P. acuta* compared to *M. capitata* in the light conditions (Fig. 6c, d; Table S4).

### Calcification rate

**Fig. 7:**
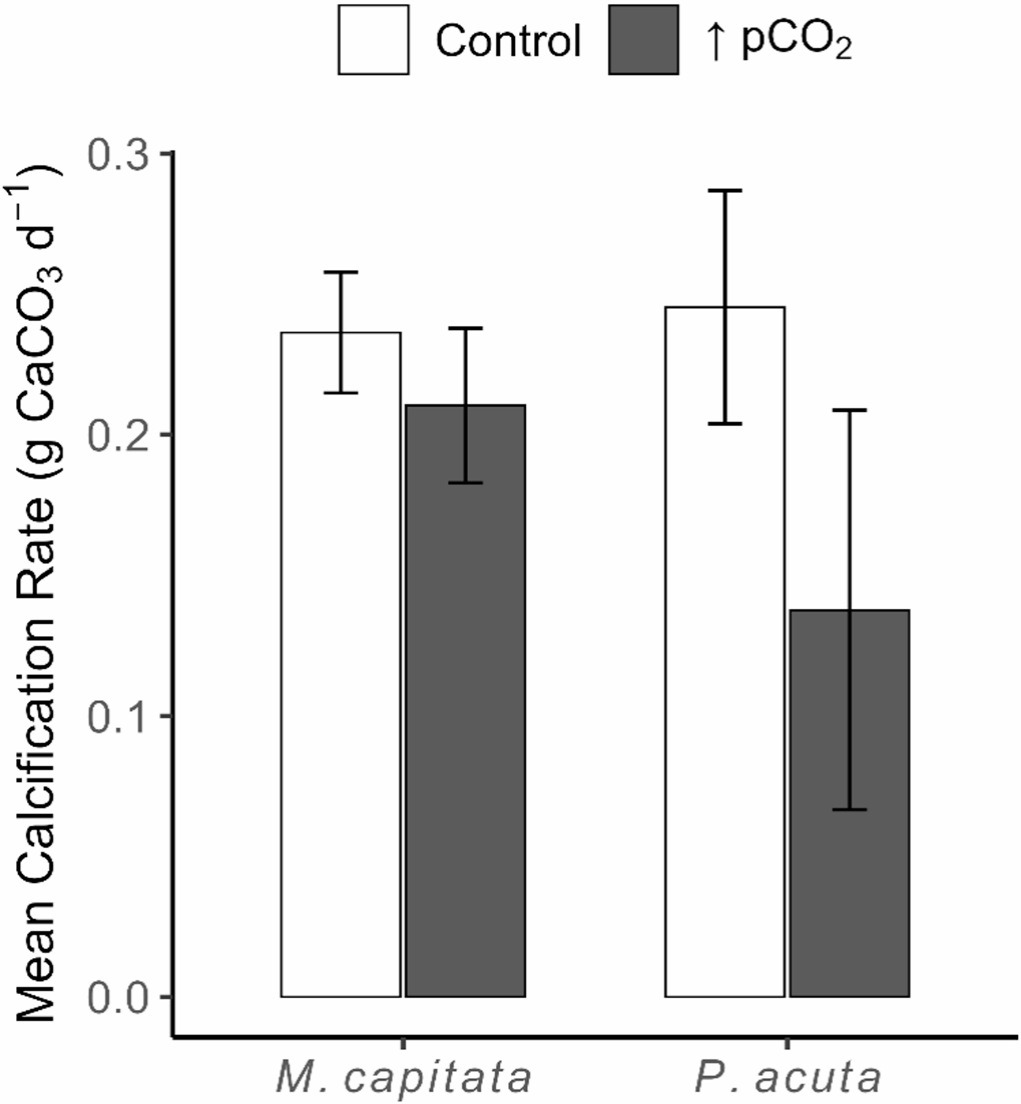
A bar plot of calcification rates in the change of buoyant weight over the 19-day exposure period. Bars represent the mean (*N* = 3) ± SE and are grouped by species, *Montipora capitata* and *Pocillopora acuta*. Bar color shows treatment where uncolored = control or ambient conditions, and grey = ↑pCO_2_ or ocean acidification conditions. There was no evidence of significant differences among the groups.

A two-way ANOVA showed no strong evidence to support a significant impact on the calcification rates of both *M. capitata* and *P. acuta*. Nevertheless, comparisons of means showed a Δ calcification rate of −0.11 g CaCO_3_ d^− 1^ in *P. acuta*, which was notably greater than *M. capitata* at a Δ calcification rate of −0.04 g CaCO_3_ d^− 1^ (Fig. 7).

## Discussion

Characterization of the CBL is essential for understanding the direct impacts of environmental stress on coral species and may explain observed responses. However, species comparisons (particularly branching small-polyped corals) seldom include CBL measurements as a response to environmental stress. In this study, CBL profile traits revealed reduced proton efflux in *P. acuta* but not *M. capitata* under elevated pCO_2_ measured in the distal and rapidly calcifying areas. Calcification data from previous studies show that *M. capitata* is more resistant to OA whereas *P. acuta* is more vulnerable^12,13,17^. Reduced dark proton efflux and thus increased interstitial [H^+^] could determine interspecific calcification sensitivities to increased seawater pCO_2_^23,26,41^. We hypothesize that: (1) reduced dark proton efflux reflects decreased calcification due to increased seawater [H^+^] ions and potentially increased interstitial [H^+^], (2) O_2_ efflux increased under light conditions due to increased photosynthetic rates, and (3) microchemical extremes within the CBL induced by species micromorphology was a major factor for interspecific responses observed.

### Proton and oxygen flux

**Fig. 8:**
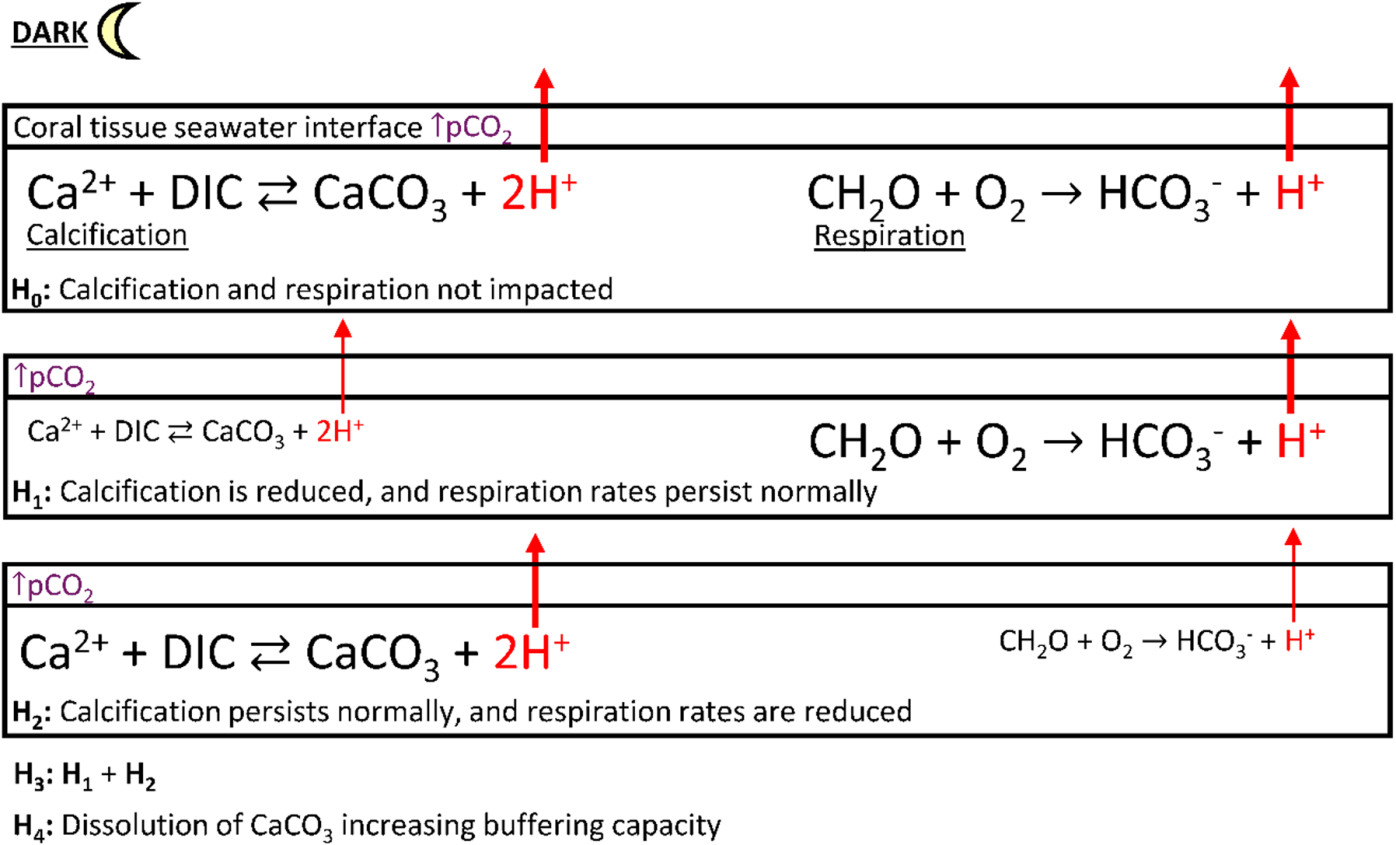
Conceptual model of hypotheses (H_0_-H_4_) for reduced proton (H^+^) efflux in dark conditions under ↑pCO_2_ = ocean acidification. The arrow and equation text size represent the magnitude of flux, where thinner arrows with smaller text indicate lower flux and thicker arrows with larger text show greater flux.

This study conducted measurements within the ZPC, which, for morphologically branching corals, is constrained to the distal tips of each branch, primarily contributing to linear extension and rapid calcification^23,41^. Material exchange (flux) between the coral organism and seawater within the ZPC is essential for maintaining the internal processes such as calcification, photosynthesis, and respiration^46,48–50^. Under elevated pCO_2,_ proton efflux in *P. acuta* was on average significantly repressed by 84% from ~ 2.5 to ~ 0.4 (µmol m^− 2^ s^− 1^ × 10^4^) in dark conditions. On the other hand, the species *M. capitata* exhibited little to no change in proton efflux under elevated pCO_2_, resulting in a non-significant Δ from ~ 0.29 to ~ 0.27 (µmol m^− 2^ s^− 1^ × 10^4^) in dark conditions. These findings suggest interspecific differences in proton flux, can be constrained and simplified into two internal processes, calcification and respiration (Fig. 8).

**Fig. 9:**
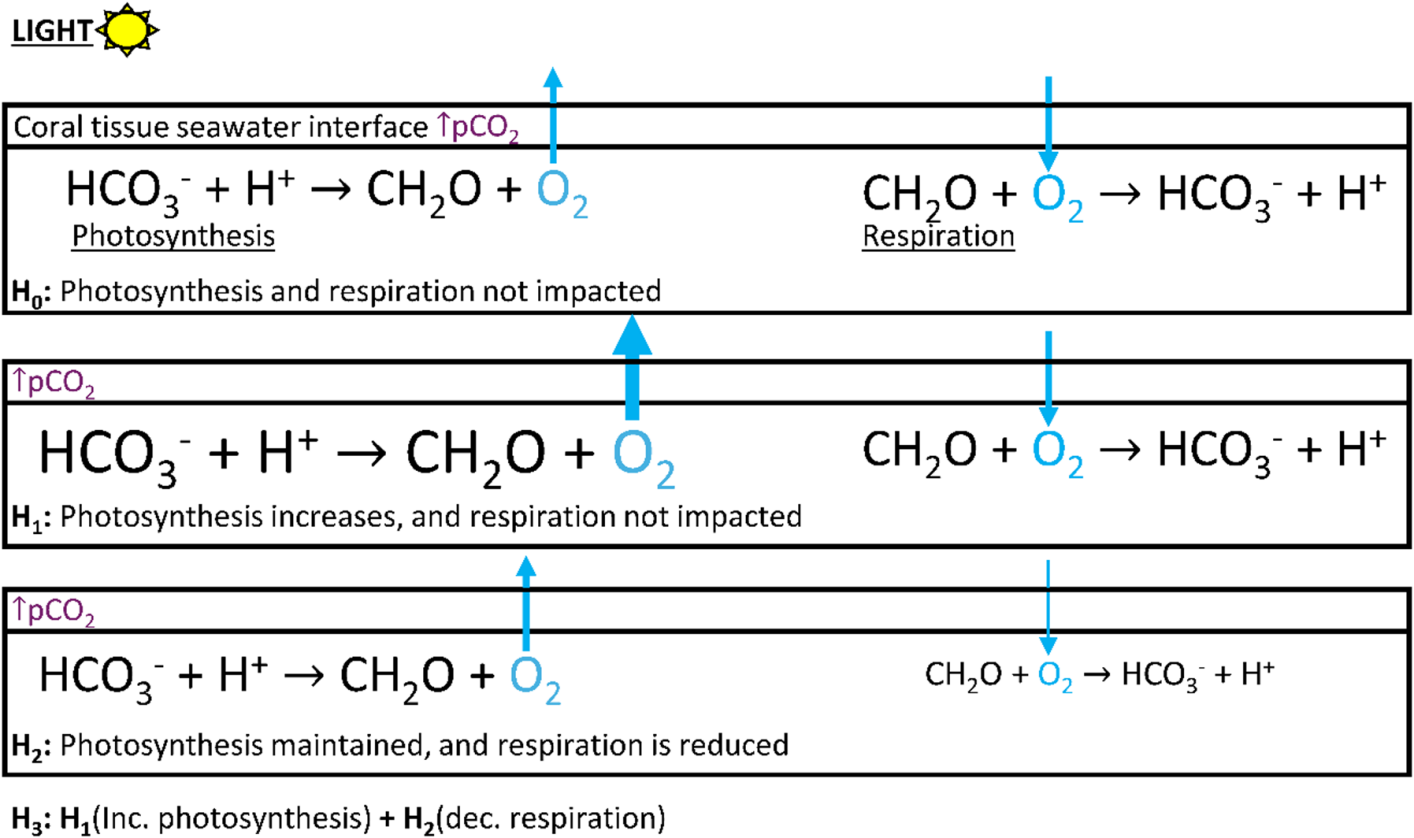
Conceptual model of hypotheses (H_0_-H_3_) for increased oxygen (O_2_) efflux in light conditions under ↑pCO_2_ = ocean acidification. The arrow and equation text size represent the magnitude of flux, where thinner arrows with smaller text indicate lower flux and thicker arrows with larger text show greater flux.

To understand the physiological processes dictating differences in dark proton efflux observed in this study, hypotheses H_0_-H_4_ were evaluated to suggest the impacts of elevated pCO_2_ (Fig. 8). A species comparison indicated that *M. capitata* aligned strongly with H_0_, whereas *P. acuta* could fall within the bounds of H_1_-H_4_. However, contrasting to Comeau et al.^51^, there was no strong evidence for decreased dark [O_2_] flux, and thus no indication of reduced dark respiration. Therefore, the results here support eliminating reduced dark respiratory rates (H_2_ and H_3_) as primarily contributing to differences in dark proton efflux (Fig. 8). This leaves H_1_ and H_4_, both of which position calcification as the primary driver of changes in dark proton efflux. Testing H_4_ independently with data collected here is not possible and has not been achieved in any previous microchemical investigations. However, increased dissolution rates would similarly correspond to decreased calcification rates, preventing net precipitation of CaCO_3_. Results in this study therefore support H_1_, proton flux driven reduced dark calcification rates, as the most likely scenario, given the decoupling of oxygen flux and the absence of reduced respiration rates. Nevertheless, oxygen flux increased significantly increased from ~ 205 to ~ 344 (µmol m^− 2^ s^− 1^) under light conditions, prompting a secondary set of postulates including respiration during the light conditions (Fig. 9).

The species *M. capitata* showed strong evidence consistent with H_0_ for O_2_ flux, shown by the conceptual model under elevated pCO_2_ (Fig. 9). However, the response observed in *P. acuta*, may follow trends in H_1_, H_2_, or H_3_ (Fig. 9). Based from the previous postulates, results suggest no indication of decreased dark respiration rates, supporting the elimination of H_2_ and H_3_ from the discussion (Fig. 9). Although this study did not measure light-enhanced dark respiration (LEDR), previous work has found no impact of elevated pCO_2_ on LEDR in the coral species *Pocillopora verrucosa*, *Porites irregularis*, Massive *Porites* spp., and *Psammocora profundacella* in Comeau et al.^51^, or *Acropora millepora* in Kaniewska et al.^52^. Given the increase of [O_2_] efflux, this suggests that photosynthesis must occur at normal or elevated rates, independently of respiration (Fig. 9). LEDR is thermodynamically enhanced by the availability of carbohydrate (CH_2_O) and would not be limited by photosynthesis. Therefore, we are inclined to reject H_2_ and H_3_ where LEDR was decreased. An increase in photosynthesis would align with findings in *P. damicornis* reported by Strahl et al.^53^, where corals were acclimated at CO_2_ seep sites, but this contrasts with the results found by Comeau et al.^51^ in an identical species comparison.

**Fig. 10:**
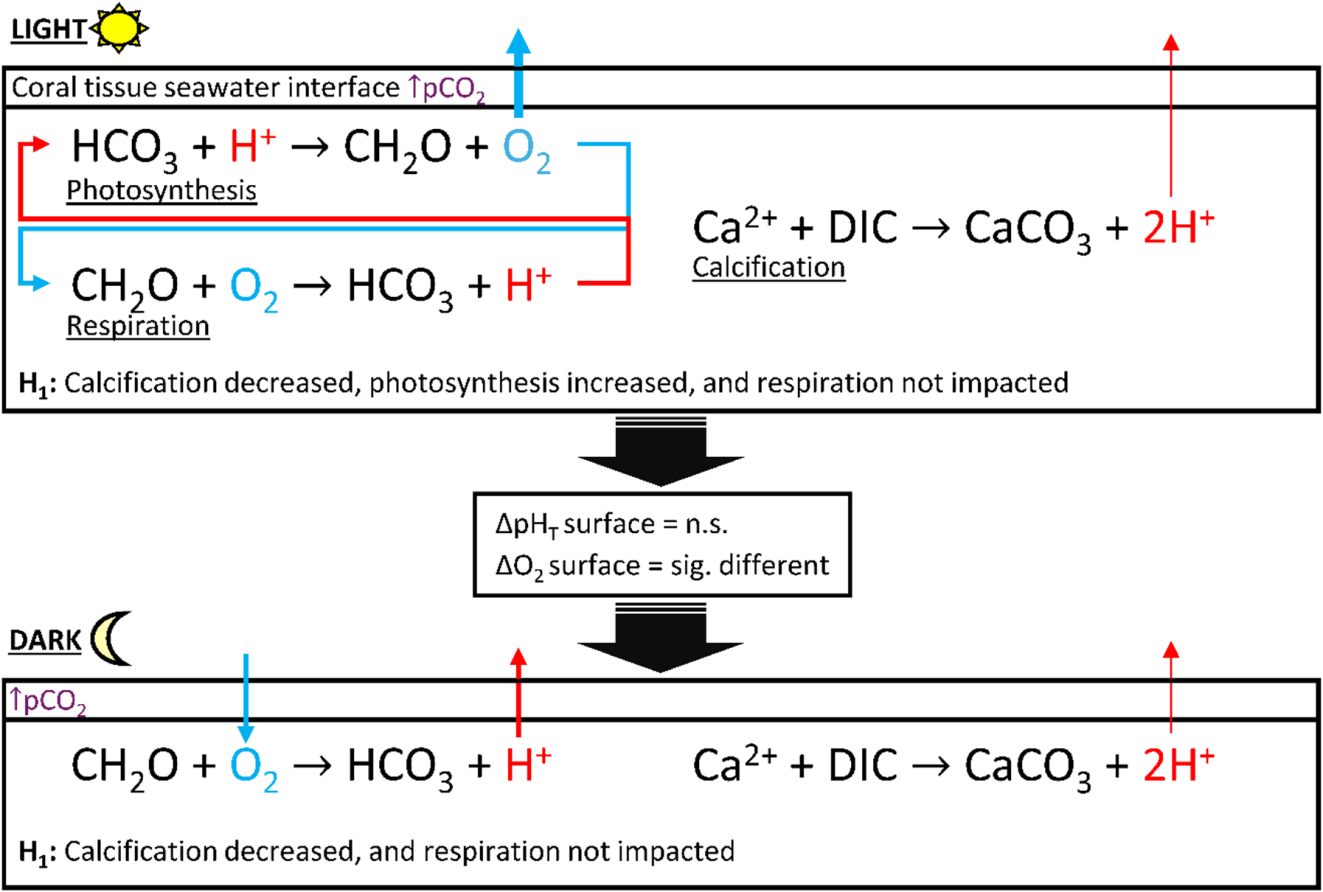
Conceptual model of final proposed hypotheses for significantly increased oxygen (O_2_) efflux in light conditions, decreased proton efflux in dark conditions, no significant difference (n.s.) in the surface of the coral—change (Δ) pH total (pH_T_), and a significant (sig.) difference the surface Δ oxygen (O_2_) concentrations under ↑pCO_2_ = ocean acidification. Arrow thickness is represents the magnitude of flux.

This study proposes that the driver of decreased calcification under elevated pCO_2_ is not metabolism (photosynthesis or respiration), but rather the ability to maintain proton efflux against a BL limitation (Fig. 10). The results in this study support a boundary layer limitation of proton flux under elevated pCO_2_^26,41^ in the distal, rapidly calcifying regions of *P. acuta* but not *M. capitata*. Comeau et al.^18^ found that pH_cf._ and its relationship with CBL seawater pH vary among species and are additionally influenced by flow and light. The work here agrees with interspecific differences in CBL response to seawater pH, but may be limited by unexplored relationships to flow and light^18,54^. Contrasting with results here, the pH of the CBL and, thus, internal pH_cf._ has been shown not to be the primary driver for reduced calcification in corals^55–57^. Nevertheless, this study demonstrated that proton flux decreased significantly under elevated pCO_2_ and proposes this result as a function of reduced calcification. However, further work is needed to determine the cf. response correlated with proton flux in *P. acuta* to better understand how the microchemical external environment may influence internal chemical gradients.

In light of the O_2_ flux findings, the results of this study contrast with those of Martins et al.^25^, who reported no effect of OA on O_2_ flux in similar *Pocilloporid* spp. under higher flow regimes than those used in the present study. Allemand et al.^58^ discussed the link between calcification and photosynthesis. They proposed a model in which carbonic anhydrases^59–61^ in the coral symbionts to fix HCO_3_^−^ as a source of DIC for photosynthesis. Their model further showed that OH^−^ produced by hydrolysis buffers H^+^ ions generated from calcification^58^. In this study, O_2_ flux increased significantly in *P. acuta* under elevated pCO_2_, which is hypothesized to have resulted from increased photosynthesis. The availability of substrates for photosynthesis increases with elevated pCO_2_^62,63^, and photosynthesis may benefit from this increased DIC pool^64^. Nevertheless, this study also observed the relative decrease in proton flux related to decreased calcification, which in turn could contribute toward reduced competition between host and algal symbionts for this shared DIC pool^65^. Future studies should continue to investigate whether elevated pCO_2_ directly stimulates photosynthesis or if reduced calcification provides less competition for DIC.

### Absolute surface oscillations

To characterize diel fluctuations of pH_T_ and O_2_ levels within the coral microenvironment, this study compared the absolute surface values under light and dark conditions at elevated pCO_2_ for both species. The results here indicated that at 0 μm (surface level), pH_T_ differed significantly between light and dark conditions only in the control group of *P. acuta*. The magnitude of pH oscillations has been shown to predict the response of corals and coral communities to acidification^66,67^. Enochs et al.^68^ demonstrated that a bulk seawater pH_T_ of 7.80 ± 0.20 produced harmful nighttime effects on calcification. This tipping point was surpassed in *P. acuta* under dark conditions and may be related to the reduction shown in proton flux.

Chan et al.^19^, showed that net coral response depends on the diel influence of pH in the microchemical environment. Greater pH oscillations result in dark extremes that may offset the benefits of elevated daytime pH. Naturally occurring high oscillations in temperature and pH have previously been shown to confer resistance to environmental change in corals^69–71^. The corals in this study were collected from a lagoonal system in Kāneʻohe Bay, Hawai’i, where natural oscillations are far greater than reefs within the archipelago (see morning and mid-day comparisons within Table 1)^72,73^. Notably, the calcification data in this study provided no compelling evidence to suggest that elevated pCO_2_ depressed bulk calcification values. Therefore, both species exhibited some degree of resistance to elevated pCO_2_, as calcification rates were nearly identical between species in the control treatments. However, *P. acuta* exhibited decreased dark proton efflux along with surface oscillations that were nearly halved compared with the control. The diminished oscillations in surface ΔpH_T,_ decoupled from ΔO_2_ under elevated pCO_2,_ suggest calcification limitations driven by seawater acidification rather than metabolism. Because *P. acuta* naturally exhibits high oscillations in ΔpH_T_ and ΔO_2,_ dark period thresholds may far exceed the tolerance limits of this coral under elevated pCO_2_.

### Interspecific properties

**Fig. 11:**
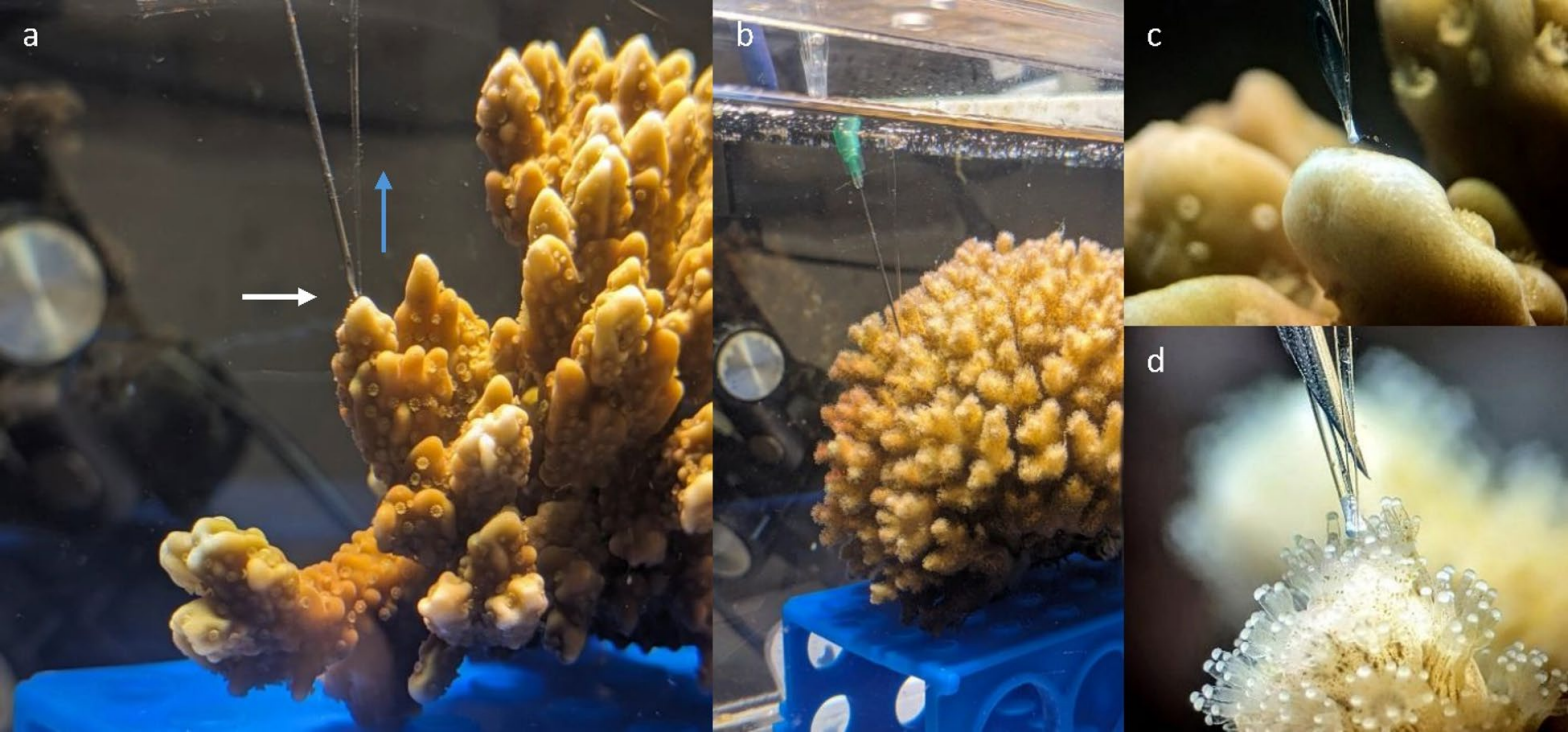
Coral colonies *M. capitata* (**a** & **c**) and *P. acuta* (**b** & **d**), stereoscope images of profile locations. The white arrow shows flow direction, and the blue arrow shows orthogonal profile direction.

Vertical profiles for both species were characterized as diffusive, complex, and S-shaped (Fig. 11a-d)^31^. However, CBL thicknesses did not differ significantly between species for either O_2_ or pH. Visibly, the profiles in *P. acuta* were highly complex or S-shaped in most cases, where notably, the microtopography of this coral species has larger and more dense polyps than *M. capitata*^34,35,74^. *Montipora* spp. also have tissue thicknesses six times thicker than those of *Pocillopora* spp. (~ 608 μm and ~ 183 μm, respectively)^75^. Ocean acidification can induce changes in microtopography, including polyp size^76^ and potentially ciliary activity^32,77^. Pacherres et al.^32^, demonstrated that ciliary vortices were essential for oxygen diffusion and flux surrounding the cenosarc. Future studies should investigate whether an increase in oxygen flux under OA is linked to a decreased capacity of cilia to diffuse oxygen. Coupling oxygen and proton flux measurements with particle image velocimetry, as applied by Pacherres et al.^32^, could elucidate cilia behavior and its relationship with external mass exchange.

In species comparisons, little has been documented regarding CBL characteristics of branching small polyp corals^25,27^. This study aimed to show CBL traits in dissimilar microtopographic complexes between macromorphologically similar species. Slow flow (1250 μm/s) produced anoxia (0.57 ± 0.08 O_2_ mg L^− 1^) in dark conditions of *P. acuta* and never reached a [O_2_] < 3.8 mg L^− 1^ in *M. capitata*. Shashar et al.^78^ similarly reported dark induced anoxia in the massive coral *Favia favus*. In the microenvironment directly above the coenosarc, O_2_ diffusion was thought to be primarily responsible for the external [O_2_]^32,77,79^. However, high ciliary action has been linked to the heterogeneous distribution of O_2_ within the CBL and correlated with reductions in oxidative stress^32,33^.

Overall, these data support the findings of Comeau et al.^18,54^ that slow flow does not provide refuge from OA, as microchemical extremes are exacerbated in the dark. Cornwall et al.^28^ argued that low flow environments resulted in daytime pH increases from photosynthesis that ameliorated OA driven pH decreases in marine calcifying macroalgae. However, our data showed that slower flow speeds created extreme microchemical and thick CBLs characterized by low pH and [O_2_] in dark conditions.

## Conclusion

This study demonstrates interspecific differences in proton and O_2_ flux under elevated pCO_2_. *P. acuta* exhibited a coupled response of decreased dark proton efflux and increased light O_2_ efflux, whereas *M. capitata* showed no detectable treatment effect. Through deductive reasoning, this study concluded that in *P. acuta*, dark calcification independent of coral metabolism was diminished under elevated pCO_2_ via reduced proton efflux from the CBL. Conversely, increased O_2_ flux under light conditions was likely attributed to increased photosynthetic rates. Simultaneous measurements of pH and O_2_ within the CBL enable the decoupling of coral metabolism and calcification. Branching, small polyped corals are seldom investigated in direct comparisons, and this is the first study to measure simultaneous proton flux coupled with O_2_ flux at the ZPC. The microchemical environment of the ZPC supports rapid linear extension and calcification, thus, reduced efflux of calcification inhibitors (protons) could threaten this process. Future studies would benefit from expanding ZPC comparisons, across a broader range of species, and pairing CBL measurements with cf. analyses such as RAMAN spectroscopy.

## Methods

### Treatment conditions

Six large colonies of *M. capitata* and *P. acuta* were collected at approximately 300 m from the shore of the Hawaiʻi Institute of Marine Biology in Kāneʻohe Bay, Hawaiʻi. Coral colonies were acclimated to a flow-through seawater mesocosm environment (an area of 1.37 m^2^ and volume ~ 50 m^3^) for ten days. Mesocosms received seawater directly from the bay, < 1 km from the collection site, with a residence time of one hour, and were environmentally controlled (e.g., temperature, dissolved oxygen, light, and all carbonate chemistry parameters were naturally adjusted)—exposed to natural light (shaded to 50% ~ PAR 800 µmol m^− 2^ s^− 1^ at solar noon). Colonies were halved and tagged via genotype and then acclimated for three days. Directly before placement in treatment conditions, the initial buoyant weight of colonies was recorded (mean W_d_ control *M. capitata*: 108 g ± 22; high pCO_2_: 109 g ± 14; *P. acuta* control: 252 g ± 28; high pCO_2_: 177 g ± 46)^80^. Coral colonies were placed in similar locations in each mesocosm for a 19-day exposure period among other coral colonies (same species) and macrophytes to control spatial acclimation differences due to the mixed flow environment.

### Seawater chemistry

**Table 1:** Mesocosm exposure tanks seawater chemistry was taken for the 19-day exposure period and shown as either morning (09:00) or mid-day (12:00) values. Values are ± SD of the mean, and (n) shows the number of independent measurements. pH values are shown in total scale (pH_T_), and A_T_ represents total alkalinity (µmol kg^− 1^). pH_T_ and A_T_ were used along with temperature (Temp) and salinity in the R package ‘*seacarb*’ to produce carbonate chemistry data (HCO_3_^−^, CO_3_^2−^, DIC, pCO_2_, and Ω_arag_). Mesocosms were naturally fed seawater from the reef in Kāneʻohe, Hawaiʻi, and to avoid swings in mid-day productivity, the flume environment used seawater from the mesocosms in the morning.

Time of Day	Treatment	Temp (°C)	Salinity	DO (mg/L)	pH_T_	A_T_ (µmol kg^-1^)	HCO_3_^-^ (µmol kg^-1^)	CO_3_^2-^ (µmol kg^-1^)	DIC (µmol kg^-1^)	pCO_2_ (µatm)	Ω_arag_
Holding Mesocosms											
Morning	Control	26.80 ± 0.18 (*n* = 6)	35.52 ± 0.05 (*n* = 6)	6.99 ± 0.17 (*n* = 6)	7.97 ± 0.03 (*n* = 6)	2179 ± 7 (*n* = 6)	1714 ± 19 (*n* = 6)	187 ± 10 (*n* = 6)	1914 ± 11 (*n* = 6)	461 ± 33 (*n* = 6)	2.99 ± 0.16 (*n* = 6)
	↑CO_2_	26.83 ± 0.23 (*n* = 6)	35.56 ± 0.06 (*n* = 6)	6.94 ± 0.12 (*n* = 6)	7.67 ± 0.05 (*n* = 6)	2178 ± 10 (*n* = 6)	1916 ± 24 (*n* = 6)	106 ± 11 (*n* = 6)	2050 ± 18 (*n* = 6)	1030 ± 126 (*n* = 6)	1.69 ± 0.18 (*n* = 6)
Mid-day	Control	27.28 ± 0.29 (*n* = 30)	35.39 ± 0.19 (*n* = 30)	7.54 ± 0.31 (*n* = 30)	8.10 ± 0.04 (*n* = 30)	2168 ± 12 (*n* = 9)	1584 ± 18 (*n* = 9)	234 ± 5 (*n* = 9)	1827 ± 15 (*n* = 9)	319 ± 11 (*n* = 9)	3.75 ± 0.07 (*n* = 9)
	↑CO_2_	27.25 ± 0.30 (*n* = 30)	35.39 ± 0.20 (*n* = 30)	7.50 ± 0.23 (*n* = 30)	7.83 ± 0.06 (*n* = 30)	2174 ± 13 (*n* = 9)	1811 ± 24 (*n* = 9)	146 ± 10 (*n* = 9)	1975 ± 18 (*n* = 9)	670 ± 59 (*n* = 9)	2.34 ± 0.16 (*n* = 9)
Flume Environment											
Morning	Control	26.58 ± 0.10 (*n* = 6)	35.55 ± 0.04 (*n* = 6)	6.86 ± 0.12 (*n* = 6)	7.96 ± 0.02 (*n* = 6)	2178 ± 10 (*n* = 6)	1722 ± 15 (*n* = 6)	184 ± 7 (*n* = 6)	1918 ± 11 (*n* = 6)	470 ± 24 (*n* = 6)	2.93 ± 0.11 (*n* = 6)
	↑CO_2_	26.13 ± 0.23 (*n* = 6)	35.61 ± 0.06 (*n* = 6)	6.81 ± 0.41 (*n* = 6)	7.66 ± 0.03 (*n* = 6)	2179 ± 7 (*n* = 6)	1927 ± 15 (*n* = 6)	102 ± 7 (*n* = 6)	2057 ± 12 (*n* = 6)	1047 ± 84 (*n* = 6)	1.62 ± 0.11 (*n* = 6)

All parameters, i.e., temperature (C^°^), salinity, and nutrients, were not manipulated from the natural seawater (Table 1). Seawater carbonate chemistry was manipulated independently via the bubbling of pure CO_2_ gas and air mixture directly into mixing pumps in each of the holding mesocosms (Table 1). A pH_stat_ methodology was implemented where pCO_2_ levels were controlled via the change in seawater pH, and this study aimed for a target ΔpH < 0.3 from control mesocosms, which was achieved through increasing or decreasing bubble rates of CO_2_ in each mesocosm^81^. However, the flume environment (Figure S1) collected water from only the control mesocosms, and CO_2_ was bubbled not directly into the flume but into 1000 mL of seawater until a pH of 4.00 units was achieved, then mixed for one hour with pumps on high until a target quasi-steady-state pH < 0.3. All total alkalinity (A_T_) measurements were conducted via acid titrations (0.1 M HCl titrant) using a Metrohm Titrino 877 Plus, and all raw A_T_ values were corrected to a certified reference material (CRM) batch #205 (bottled 09/09/2022; Table 1)^82^. Measurements of temperature, salinity, dissolved oxygen (DO mg L^− 1^), and pH_NBS_ were collected using a YSI ProDDS Multiparameter Digital Water Quality Meter, and subsequent pH_NBS_ values were corrected to a Tris Buffer 41 solution to achieve pH_T_.

### Flume environment

The flume was constructed using cell-cast acrylic formed into two sections, the concentrator (2.09 m^2^) and the test Sect. (88.9 × 22.3 cm; 1.8 m^2^; Figure S1). Water flowed into the concentrator via a pump (24 V DC Hygger – 6511 LPH) to a spray bar manifold in the concentrator section. Water was directed to a flow-reducing baffle and exited the concentrator (1250 μm s^− 1^; τ = 44 min) through a hexagonal flow straightener separating the concentrator and test section. Seawater then exited at the end of the test section through standpipes and gravity-fed into a reservoir below. A 180-w, Wattshine full-spectrum LED aquarium light was affixed above the test section (PAR 800 µmol s^− 1^), and a micromanipulator arm extended over the test section.

### Microsensors and manipulator movements

The O_2_ microsensor was a PreSens micro-optode (PSt7 – Flat Broken Tip) at a diameter of 230 μm. The pH microsensor is a Unisense microelectrode with a tapered glass tip diameter of 100 μm. The microsensors recorded continuously (1 measurement every 6 s) on respective proprietary programs for the duration of the profile. Sensors were both mounted in a dual-probe auto-micromanipulator (Zaber T-LSR075A) and synced with programming software provided by Zaber. Profiles were conducted where the movement was directed via scripts delivered directly to the auto-micromanipulator, with two separate scripts switched by the user. The sensors were set to pause for 60 s at each height and continue to record continuously with one measurement every six seconds. For the duration the sensor was at a specified height, ten measurements were collected for each parameter and were averaged to characterize each height. The stage was set to travel at 5 μm s^− 1^ to each specified height, and these processes were repeated until the script finished. From the surface (0 μm), this regime repeated every 100 μm until subsequently reaching a value that reflected the bulk concentration or 2500 μm. Once a height where measurements of O_2_ and pH reflected the bulk seawater values, the micromanipulator was set to continue an extra 2000 μm at 500 μm intervals to obtain tailing bulk measurements outside of the CBL. All measurements of O_2_ and pH were standardized to bulk values taken via the external probes on the YSI of the flume seawater at the time of profiling^38,83^.

### Point selection on colonies

Microsensors were positioned according to set parameters that remained constant throughout every profile. O_2_ sensors were directly beside pH sensors and manually moved as close as possible to the human eye when viewing through a stereoscope. Sensor positioning will follow these criteria in every profile: Locating a ‘tip’ section directly facing into the semi-straight water flow, positioned ‘between polyps,’ and lowering the sensors at micro-scale increments (1–10 μm) until parameters of O_2_ and pH reach their maximum at a quasi-steady-state (Fig. 11a-d).

### CBL thickness

The CBL thickness (δCBL) was calculated by fitting a linear model to the log-transformed concentration profile, expressed as log_10_([X]) as a function of distance (*x*) from the coral surface (Eq. 1). Using the fitted intercept (*α*) and slope (*β*), we then extrapolated the distance at which the concentration reached the independently measured bulk concentration threshold ([X]_bulk_) (Eq. 2).

1 $${\text{lo}}{{\text{g}}_{10}}\left( {\left[ {\text{X}} \right]\left( x \right)} \right){\text{ }}=\alpha \,+\,\beta x$$

2 $$\delta {\text{CBL}}\,=\,{\text{lo}}{{\text{g}}_{10}}\left( {{{\left[ {\text{X}} \right]}_{{\text{bulk}}}}} \right)-\alpha /\beta$$

### Proton and oxygen flux

Both proton and oxygen flux values were calculated using the H^+^ and O_2_ diffusion coefficient, following the methods described in detail in Pacherres et al.^33^ and replicated from Martins et al.^25,31^ using the upper linear gradient of complex profiles. Proton flux (*J*_*H*_^*+*^) was calculated using Fick’s first law of diffusion, with a linear model applied to the proton concentration (*X*_*H*_^*+*^) profile across the distance (*x*) to determine the concentration gradient (*m*). The diffusion coefficient for protons (D_H_^+^) at a salinity of 35 and a temperature of 26.5 °C (Eq. 3).

3 $${J_H}^{+}= - (9.31 \times {10^{ - \,5}}) \cdot m$$

Where *J*_*H*_^*+*^ is the proton flux, D_H_^+^ = 9.31 × 10^− 5^ cm^2^ s^− 1^, and *m* is the slope (gradient) from the linear model of *X*_*H*_^*+*^ as a function of distance.

Oxygen flux (*J*_*O2*_​​) was determined using Fick’s first law of diffusion. A linear model was applied to the oxygen concentration (*X*_*O2*_​) profile across distance to calculate the concentration gradient (*m*), with the diffusion coefficient for oxygen (*D*_*O2*_​​) at a salinity of 35 and temperature of 26.5 °C set at 2.20 × 10^− 5^ cm s^− 1^ (Eq. 4).

4 $${J_{O2}}= - (2.20 \times {10^{ - \,5}}) \cdot m$$

Where *J*_*O2*_ ​​ is the oxygen flux, *D*_*O2*_ = 2.20 × 10^− 5^ cm^2^ s^− 1^, and *m* is the slope (gradient) from the linear model of *X*_*O2*_​​ as a function of *d*.

### Statistics

To assess the effects of species, treatment, and condition on proton and oxygen flux, we used a series of linear mixed-effects models (LMMs) and linear models (LMs) in R (v4.2.0)^84^ with the ‘*lme4*’ and ‘*emmeans*’ packages. The initial model included all interactions between species, treatment, and condition, along with a random effect for genotype. AIC was used to compare models, and genotype was excluded in several instances due to minimal impact on model fit. The final model, which included all interaction terms without the random effect for genotype, was selected based on the lowest AIC. Residual diagnostics confirmed model assumptions. Post-hoc analyses using estimated marginal means and Bonferroni-adjusted pairwise comparisons identified significant differences among treatment, species, and condition combinations. All statistical analyses were conducted at a significance level of α = 0.05 (R Core Team).

## Supplementary Information

Below is the link to the electronic supplementary material.

Supplementary Material 1

## Data Availability

All raw data and source code used in the analysis are publicly available and found here at https://github.com/CROH-Lab/Proton_and_oxygen_flux_concentration_boundary_layer.git.
