# Supplementary material for: Ocean acidification modulates material flux linked with coral calcification and photosynthesis
Source: Sci Rep. 2025 Dec 12;16:1255. doi: 10.1038/s41598-025-30818-4 (PMC12789429; doi:10.1038/s41598-025-30818-4)
Supplement: Supplementary file 1 — Supplementary Material 1 [file 41598_2025_30818_MOESM1_ESM.docx]

**Supplementary information**

**Table S1.** Significant p-value results from transformed pairwise comparison of interactions between treatment, condition, and species to proton flux.

| Group A | Group B | Δ (YJ scale) | SE | df | t | p (Bonferroni) |
| --- | --- | --- | --- | --- | --- | --- |
| *P. acuta* - control - Dark | *P. acuta* - ↑ pCO₂ - Light | 2.09e-04 | 2.17e-05 | 16 | 9.62 | <0.0001 |
| *P. acuta* - control - Dark | *M. capitata* - ↑ pCO₂ - Light | 1.38e-04 | 2.17e-05 | 16 | 6.34 | 0.0003 |
| *P. acuta* - control - Dark | *P. acuta* - control - Light | 1.31e-04 | 2.17e-05 | 16 | 6.02 | 0.0005 |
| *P. acuta* - control - Dark | *M. capitata* - control - Light | 1.28e-04 | 2.17e-05 | 16 | 5.88 | 0.0007 |
| *P. acuta* - ↑ pCO₂ - Dark | *P. acuta* - ↑ pCO₂ - Light | 1.27e-04 | 2.17e-05 | 16 | 5.86 | 0.0007 |
| *M. capitata* - control - Dark | *P. acuta* - ↑ pCO₂ - Light | 1.21e-04 | 2.17e-05 | 16 | 5.56 | 0.0012 |
| *M. capitata* - ↑ pCO₂ - Dark | *P. acuta* - ↑ pCO₂ - Light | 1.19e-04 | 2.17e-05 | 16 | 5.47 | 0.0014 |
| *P. acuta* - control - Dark | *M. capitata* - ↑ pCO₂ - Dark | 9.01e-05 | 2.17e-05 | 16 | 4.14 | 0.0213 |
| *M. capitata* - control - Dark | *P. acuta* - control - Dark | -8.81e-05 | 2.17e-05 | 16 | -4.05 | 0.0259 |
| *P. acuta* - control - Dark | *P. acuta* - ↑ pCO₂ - Dark | 8.18e-05 | 2.17e-05 | 16 | 3.76 | 0.0478 |
| *M. capitata* - control - Light | *P. acuta* - ↑ pCO₂ - Light | 8.14e-05 | 2.17e-05 | 16 | 3.74 | 0.0497 |

**Table S2.** Significant p-value results from transformed pairwise comparison of interactions between treatment, condition, and species to oxygen flux.

| Group A | Group B | Δ (response scale) | SE | df | t | p (Bonferroni) |
| --- | --- | --- | --- | --- | --- | --- |
| *P. acuta* - control - Dark | *P. acuta* - ↑ pCO₂ - Light | -508 | 35.4 | 16 | -14.40 | <0.0001 |
| *P. acuta* - ↑ pCO₂ - Dark | *P. acuta* - ↑ pCO₂ - Light | -482 | 35.4 | 16 | -13.60 | <0.0001 |
| *M. capitata* - control - Dark | *P. acuta* - ↑ pCO₂ - Light | -375 | 35.4 | 16 | -10.60 | <0.0001 |
| *P. acuta* - control - Dark | *P. acuta* - control - Light | -369 | 35.4 | 16 | -10.40 | <0.0001 |
| *M. capitata* - ↑ pCO₂ - Dark | *P. acuta* - ↑ pCO₂ - Light | -368 | 35.4 | 16 | -10.40 | <0.0001 |
| *P. acuta* - ↑ pCO₂ - Dark | *P. acuta* - control - Light | -342 | 35.4 | 16 | -9.68 | <0.0001 |
| *P. acuta* - control - Dark | *M. capitata* - control - Light | -284 | 35.4 | 16 | -8.03 | <0.0001 |
| *M. capitata* - ↑ pCO₂ - Light | *P. acuta* - ↑ pCO₂ - Light | -267 | 35.4 | 16 | -7.56 | <0.0001 |
| *P. acuta* - ↑ pCO₂ - Dark | *M. capitata* - control - Light | -257 | 35.4 | 16 | -7.28 | <0.0001 |
| *P. acuta* - control - Dark | *M. capitata* - ↑ pCO₂ - Light | -241 | 35.4 | 16 | -6.81 | 0.0001 |
| *M. capitata* - control - Dark | *P. acuta* - control - Light | -236 | 35.4 | 16 | -6.67 | 0.0002 |
| *M. capitata* - ↑ pCO₂ - Dark | *P. acuta* - control - Light | -229 | 35.4 | 16 | -6.46 | 0.0002 |
| *M. capitata* - control - Light | *P. acuta* - ↑ pCO₂ - Light | -224 | 35.4 | 16 | -6.34 | 0.0003 |
| *P. acuta* - ↑ pCO₂ - Dark | *M. capitata* - ↑ pCO₂ - Light | -214 | 35.4 | 16 | -6.06 | 0.0005 |
| *M. capitata* - control - Dark | *M. capitata* - control - Light | -151 | 35.4 | 16 | -4.27 | 0.0164 |
| *M. capitata* - ↑ pCO₂ - Dark | *M. capitata* - control - Light | -144 | 35.4 | 16 | -4.06 | 0.0254 |
| *P. acuta* - control - Dark | *M. capitata* - ↑ pCO₂ - Dark | -140 | 35.4 | 16 | -3.96 | 0.0312 |
| *P. acuta* - control - Light | *P. acuta* - ↑ pCO₂ - Light | -139 | 35.4 | 16 | -3.94 | 0.0330 |
| *M. capitata* - control - Dark | *P. acuta* - control - Dark | 133 | 35.4 | 16 | 3.75 | 0.0484 |

**Table S3.** Significant p-value results from transformed pairwise comparison of interactions between treatment, condition, and species to pH_T_ values at the surface of the coral.

| Group A | Group B | Δ (response scale) | SE | df | t | p (Bonferroni) |
| --- | --- | --- | --- | --- | --- | --- |
| *P. acuta* - control - Dark | *P. acuta* - control - Light | -1.030 | 0.132 | 14 | -7.75 | <0.0001 |
| *P. acuta* - control - Dark | *M. capitata* - control - Light | -0.958 | 0.132 | 14 | -7.24 | 0.0001 |
| *P. acuta* - control - Dark | *P. acuta* - ↑ pCO₂ - Light | -0.912 | 0.132 | 14 | -6.89 | 0.0002 |
| *P. acuta* - control - Dark | *M. capitata* - ↑ pCO₂ - Light | -0.724 | 0.132 | 14 | -5.46 | 0.0023 |
| *M. capitata* - ↑ pCO₂ - Dark | *P. acuta* - control - Light | -0.670 | 0.132 | 14 | -5.06 | 0.0049 |
| *M. capitata* - ↑ pCO₂ - Dark | *M. capitata* - control - Light | -0.602 | 0.132 | 14 | -4.54 | 0.0129 |
| *P. acuta* - ↑ pCO₂ - Dark | *P. acuta* - control - Light | -0.577 | 0.132 | 14 | -4.36 | 0.0184 |
| *M. capitata* - ↑ pCO₂ - Dark | *P. acuta* - ↑ pCO₂ - Light | -0.555 | 0.132 | 14 | -4.19 | 0.0253 |
| *M. capitata* - control - Dark | *P. acuta* - control - Dark | 0.517 | 0.132 | 14 | 3.90 | 0.0449 |
| *M. capitata* - control - Dark | *P. acuta* - control - Light | -0.510 | 0.132 | 14 | -3.85 | 0.0492 |

**Table S4.** Significant p-value results from transformed pairwise comparison of interactions between treatment, condition, and species to [O_2_] mg L^-1^ measured at the surface of the coral.

| Group A | Group B | Δ (response scale) | SE | df | t | p (Bonferroni) |
| --- | --- | --- | --- | --- | --- | --- |
| *P. acuta* - control - Dark | *P. acuta* - ↑ pCO₂ - Light | -16.80 | 1.18 | 14 | -14.20 | <0.0001 |
| *P. acuta* - ↑ pCO₂ - Dark | *P. acuta* - ↑ pCO₂ - Light | -15.30 | 1.18 | 14 | -12.90 | <0.0001 |
| *P. acuta* - control - Dark | *P. acuta* - control - Light | -13.80 | 1.18 | 14 | -11.70 | <0.0001 |
| *M. capitata* - ↑ pCO₂ - Dark | *P. acuta* - ↑ pCO₂ - Light | -12.50 | 1.18 | 14 | -10.60 | <0.0001 |
| *M. capitata* - control - Dark | *P. acuta* - ↑ pCO₂ - Light | -12.30 | 1.18 | 14 | -10.40 | <0.0001 |
| *P. acuta* - ↑ pCO₂ - Dark | *P. acuta* - control - Light | -12.20 | 1.18 | 14 | -10.30 | <0.0001 |
| *P. acuta* - control - Dark | *M. capitata* - control - Light | -11.30 | 1.18 | 14 | -9.57 | <0.0001 |
| *P. acuta* - control - Dark | *M. capitata* - ↑ pCO₂ - Light | -11.00 | 1.18 | 14 | -9.29 | <0.0001 |
| *P. acuta* - ↑ pCO₂ - Dark | *M. capitata* - control - Light | -9.75 | 1.18 | 14 | -8.25 | <0.0001 |
| *M. capitata* - ↑ pCO₂ - Dark | *P. acuta* - control - Light | -9.48 | 1.18 | 14 | -8.02 | <0.0001 |
| *P. acuta* - ↑ pCO₂ - Dark | *M. capitata* - ↑ pCO₂ - Light | -9.41 | 1.18 | 14 | -7.96 | <0.0001 |
| *M. capitata* - control - Dark | *P. acuta* - control - Light | -9.30 | 1.18 | 14 | -7.86 | <0.0001 |
| *M. capitata* - ↑ pCO₂ - Dark | *M. capitata* - control - Light | -7.02 | 1.18 | 14 | -5.94 | 0.0010 |
| *M. capitata* - control - Dark | *M. capitata* - control - Light | -6.84 | 1.18 | 14 | -5.78 | 0.0013 |
| *M. capitata* - ↑ pCO₂ - Dark | *M. capitata* - ↑ pCO₂ - Light | -6.68 | 1.18 | 14 | -5.65 | 0.0017 |
| *M. capitata* - control - Dark | *M. capitata* - ↑ pCO₂ - Light | -6.50 | 1.18 | 14 | -5.50 | 0.0022 |
| *M. capitata* - ↑ pCO₂ - Light | *P. acuta* - ↑ pCO₂ - Light | -5.84 | 1.18 | 14 | -4.94 | 0.0061 |
| *M. capitata* - control - Light | *P. acuta* - ↑ pCO₂ - Light | -5.50 | 1.18 | 14 | -4.66 | 0.0104 |


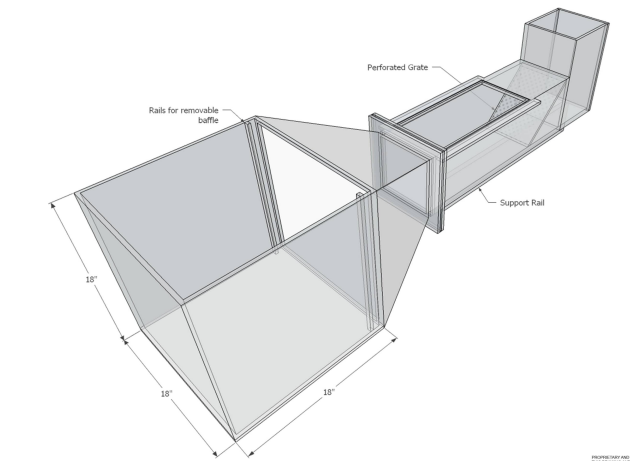


**Figure S1**. The flume environment with the concentrator defined as the section with a removable baffle to reduce turbulence, and the test section as the elongated section that is affixed via the acrylic flange. Corals were tested in the test section in the middle between the perforated grate and concentrator flange.
